# Supplementary material for: Patterns of Intron Gain and Loss in Fungi
Source: PLoS Biol. 2004 Nov 30;2(12):e422. doi: 10.1371/journal.pbio.0020422 (PMC532390; doi:10.1371/journal.pbio.0020422)
Supplement: Table S1 — Also available at http://genes.mit.edu/NielsenEtAl/. (4.3 MB ZIP). [file pbio.0020422.st001.zip › NielsenEtAl/html/580.html]

AN2970.1.NCU00088.1.MG10149.1.FG06718.1


```
 CLUSTAL W (1.82) Multiple Sequence Alignments - Introns Inserted


Sequence 1: MG10149.1	286 aa
Sequence 2: FG06718.1	292 aa
Sequence 3: NCU00088.1	306 aa
Sequence 4: AN2970.1	282 aa
Alignment Length: 311 aa
Number Identitical Residues: 126 aa
Alignment Score (without introns) 6927


MG10149.1 	MAQTQTSQFLSGNTAAINEFLDKFD0TFLLDCD-~----------------------D1K
NCU00088.1	MAQ---PKYLSGDVAAINEFIDKFD0VFLFDCDG~VLWSGEHVFEGVVETLELLRSRG1K
FG06718.1 	MAS--SPKYISNDVAAINEFIDKFD0VFLLDCDG1VLWSGDHVYEGVPETINFLRSKG1K
AN2970.1  	MAMT-VPRYLTGDPAGIKEFLDKFD0VFLFDCDG1-----------------------~-
          	** :  .::::.: *.*:**:**** .**:***.                          

MG10149.1 	RTVFVTNNSTKSRPEYQKKLASKG~IECDVDDIFGSAYSAAIYISRILKPEAPRNKVFVV
NCU00088.1	KTVFVTNNSTKSRPEYKKKFEGLG~IPADEDEIFGSAYSSAIYISRILKLAPPKNKVFVI
FG06718.1 	RVVFVTNNSTKSRDEYLKKLTGLG~IPSEKDDVFGSSYSAAIYIARILKLPEGKRKVFII
AN2970.1  	-VLWSGDHLFPGTVETLELLRSRG1-PNPKEEIFSSSYSASIYISRILNLPANKRKVFVL
          	 .::  ::   .  *  : : . *      :::*.*:**::***:***:    :.***::

MG10149.1 	GERGIEDELRSEGVPFIGGTDPAYRHDMTDADWKGLADG--SVLDPDVGVVLAGLDFHIN
NCU00088.1	GEAGIEQELTTENIPFIGGTDPSFRRDVTVEDFNGLADG--SLLNPEVGCVLVGLDWHIN
FG06718.1 	GESGIEQELDSEGVPHIGGTEEAFRRDITNDDFKGIADG--SLLDPEVGAVLCGLDYHVN
AN2970.1  	GETGIEQELRSENVPFIGGTDPSYRRDITAEDYKLIAAGDESLLDPEVGVVLVGLDFHLN
          	** ***:** :*.:*.****: ::*:*:*  *:: :* *..*:*:*:** ** ***:*:*

MG10149.1 	YLKIAHAYQYLRRGAAFIASNTDSTLPMSGNFFPGAGSTMIPIANMIGRQPLALGKPSQA
NCU00088.1	YLKLSHAYQYLRRGAVFLATNVDSTFPMNNNFFPGAGSISVPLVHMTGQEPVALGKPSQA
FG06718.1 	YLKYAHAMHYVKRGATFLATNVDSTLPMHHNFFLGAGSCHIPVVHATGQQPLALGKPSQA
AN2970.1  	YLKLALAYHYIRRGAVFLATNIDSTLPNSGTLFPGAGTVSAPLILMVGRDPVALGKPNQA
          	*** : * :*::***.*:*:* ***:*   .:* ***:   *:    *::*:*****.**

MG10149.1 	MMDAVEGKFKLDRSRTCMIGDRLDTDIKFGIEGKLGGTLAVQTGVNKKEDWEKEDAVA~R
NCU00088.1	MMDAIEGKFHLDRARTCMVGDRLNTDIKFGIEGRLGGTLAVLTGVNKKEDWEAADAVA~V
FG06718.1 	MMDAVEGKFQLDRARTCMVGDRLNTDIKFGIEGKLGGTLHVLTGVNKKEDWEKEDAIA0R
AN2970.1  	MMDAIEGKFQLDRARACMVGDRANTDIRFGLEGNLGGTLGVLTGVSSKEDFVEG--VV~R
          	****:****:***:*:**:*** :***:**:**.***** * ***..***:     :.  

MG10149.1 	PAFYVDKLSDLLAAGQ
NCU00088.1	PSFYVDKLSDLRAAAQ
FG06718.1 	S---------------
AN2970.1  	PSAYLDKLSDLLEAAE
          	.:   .. :.   :..
```
